# Pathogenicity of *Salmonella enterica* in *Caenorhabditis elegans* Relies on Disseminated Oxidative Stress in the Infected Host

**DOI:** 10.1371/journal.pone.0045417

**Published:** 2012-09-28

**Authors:** XiaoHui Sem, Mikael Rhen

**Affiliations:** Department of Microbiology, Tumor and Cell Biology, Karolinska Institutet, Stockholm, Sweden; Indian Institute of Science, India

## Abstract

Feeding *Caenorhabditis elegans* with *Salmonella enterica* serovar Typhimurium significantly shortens the lifespan of the nematode. *S*. Typhimurium-infected *C. elegans*, stained with 2′,7′-dichlorodihydrofluorescein diacetate which fluoresces upon exposure to reactive oxygen species, revealed intestinal luminal staining that along with the time of infection progressed to a strong staining in the hypodermal tissues of the nematode. Still, we could not detect invasion beyond the nematode's intestinal epithelium at any stage of the infection. A similar dispersion of oxidative response was also noted in nematodes infected with *S*. Dublin, but not with non-pathogenic *Escherichia coli* or the defined pathogen *Burkholderia thailandensis*. Addition of catalase or the reductant ascorbic acid significantly restored the lifespan of *S*. Typhimurium-infected nematodes. Mutational inactivation of the bacterial thioredoxin 1 resulted in total ablation of the hypodermal oxidative response to infection, and in a strong attenuation of virulence. Virulence of the thioredoxin 1 mutant was restored by *trans*-complementation with redox-active variants of thioredoxin 1 or, surprisingly, by exposing the thioredoxin 1 mutant to sublethal concentrations of the disulphide catalyst copper chloride prior to infection. In summary, our observations define a new aspect in virulence of *S. enterica* that apparently does not involve the classical invasive or intracellular phenotype of the pathogen, but that depends on the ability to provoke overwhelming systemic oxidative stress in the host through the redox activity of bacterial thioredoxin 1.

## Introduction

*Salmonella enterica* serovar Typhimurium (*S*. Typhimurium) is a Gram-negative enterobacterium capable of colonizing and causing infections in a wide variety of organisms ranging from Man and mice to nematodes, amoebae and plants [1]–[4]. It thereby follows that salmonellae must be equipped with virulence strategies, which allow infection of such a broad evolutionary range of hosts [5]. Still, most of the details that govern *Salmonella* infections have been obtained from murine infection models.

Upon oral infection of mice, *S*. Typhimurium invades the small intestine [6], [7], followed by dissemination to the mesenteric lymph nodes, and eventually to the liver and spleen [8]. Subsequently, the bacterium acts as a facultative intracellular pathogen and replicates in macrophages [9]–[11]. This complex infection cycle relies on several sets of virulence genes, many of which are contained on horizontally acquired genetic inserts termed *Salmonella* pathogenicity islands, or SPIs [12]. SPI1 and SPI2 code for two separate type III protein secretion systems (T3SS). With the aid of the T3SS, *S*. Typhimurium injects effector proteins into host cells; SPI1 effector proteins orchestrate actin reorganization in epithelial cells, leading to internalization and translocation of *S*. Typhimurium at the intestinal lining [13], while SPI2 effector proteins manipulate vesicular trafficking in infected cells, allowing the bacteria to replicate intracellularly [14].

SPI1 and SPI2 do not act independently from the rest of the chromosome. The highly regulated expression of the SPIs, for example, is intimately linked to many evolutionarily conserved gene regulatory systems, such as the sensor regulator systems EnvZ-OmpR and PhoP-PhoQ [15], [16]. In addition, the intracellular induction of SPI2 strongly relies on the highly conserved bacterial cytoplasmic reductase thioredoxin 1 (TrxA, *trxA*) [17]. Thus mutations in selected “house-keeping” genes, such as *trxA*, result in attenuations that equal SPI2 mutants in the murine infection model [17], [18].

The soil nematode *Caenorhabditis elegans* has been used as a biological model to study several aspects of vertebrate biology, including signal transduction, neuronal development, behavioral responses, senescence and innate immunity [19]–[21]. The high genetic tractability and large number of available tools have made *C. elegans* an emerging model to dissect the molecular basis of many mammalian diseases [22]. Furthermore, a large number of mammalian bacterial species [23], including *S*. Typhimurium , is pathogenic to the nematode. Interestingly, the EnvZ-OmpR and PhoP-PhoQ regulatory systems, as well as the SPI1 T3SS, contribute to the virulence of *S*. Typhimurium in *C. elegans* [2], [3], [24]. The SPI1 T3SS appears to generate pro-apoptotic signals in germline cells outside the intestine [24]. However, the mechanisms by which *S*. Typhimurium elicits death in *C. elegans* have not been fully clarified.

Production of reactive oxygen species (ROS) represents one of the most primordial innate defense mechanisms against many invading microbes [25]–[28]. Furthermore, lack of an efficient *phox*-mediated oxidative burst response sensitizes both Man and mice to infections with *Salmonella* spp. [29]–[31]. On the other hand, overwhelming ROS production has been implicated as a crucial pathological effector during septic shock in Man and other animals [32]–[34]. *C*. *elegans* also has the ability to mount a protective oxidative response upon infection with pathogens such as *Enterococcus faecalis* [35], [36]. In this study, we sought to elucidate the pathogenesis of *S*. Typhimurium infection in *C. elegans*. We showed that *S*. Typhimurium kills the nematode by inducing a disseminated and overwhelming host oxidative response that depends on active bacterial protein synthesis and the bacterial TrxA. Importantly, our data thereby also suggest a novel and unique aspect of TrxA's role in *S*. Typhimurium.

## Results

### *S*. Typhimurium perturbs intestinal morphology but does not disseminate beyond the intestinal border

**Figure 1:**
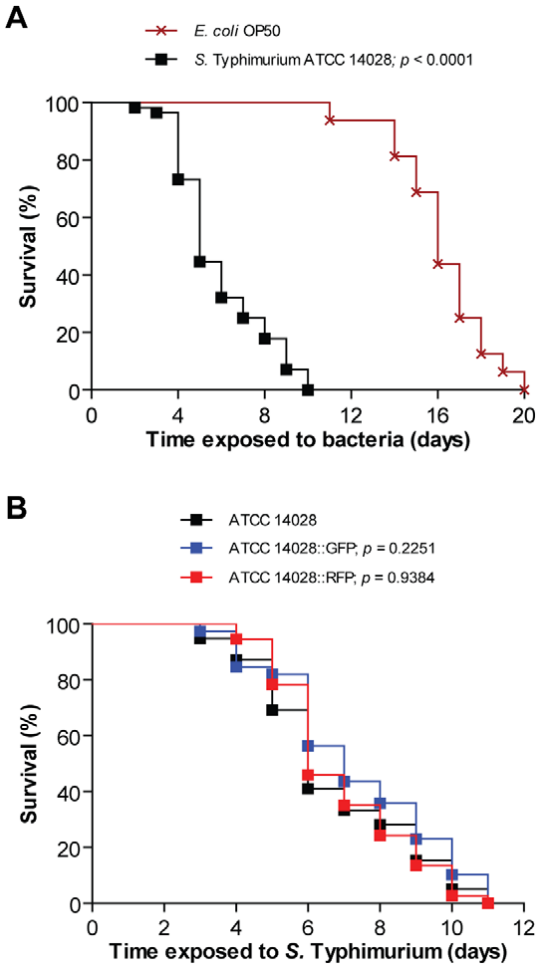
*S*. Typhimurium strain 14028 is pathogenic to *C. elegans*. (A) Survival of wild-type N2 nematodes was compared when fed *E. coli* OP50 or *S*. Typhimurium 14028 (*p*<0.0001). (B) Survival of nematodes was compared when infected with non-fluorescent *S*. Typhimurium 14028, 14028::GFP (*p* = 0.2251) or 14028::RFP (*p* = 0.9384; all *p* values as compared to the non-fluorescent strain). Each survival curve is representative of 3 independent assays, each with 3 plates per strain and 20 nematodes per plate.

When we compared the lifespans of wild-type N2 *C. elegans* propagated either on *Escherichia coli* strain OP50 or on the virulent *S*. Typhimurium strain 14028, we noted a pathogenicity of *S*. Typhimurium as evidenced by a significantly shortened lifespan (*p*<0.0001, Figure 1A), consistent with previous reports.

In murine models for typhoid fever, virulence of *S.* Typhimurium depends on its ability to invade the intestinal epithelium and subsequent dissemination to the liver and spleen. Expectedly, non-invasive mutants of *S*. Typhimurium are avirulent in mice challenged orally [37]. To test if *S*. Typhimurium pathogenicity in *C. elegans* also depends on intestinal invasion, we infected wild-type *C. elegans* with a GFP-expressing strain of *S*. Typhimurium 14028, shown to have comparable virulence as the non-fluorescent strain in mice [38] as well as in the nematode (*p* = 0.2251, Figure 1B).

**Figure 2:**
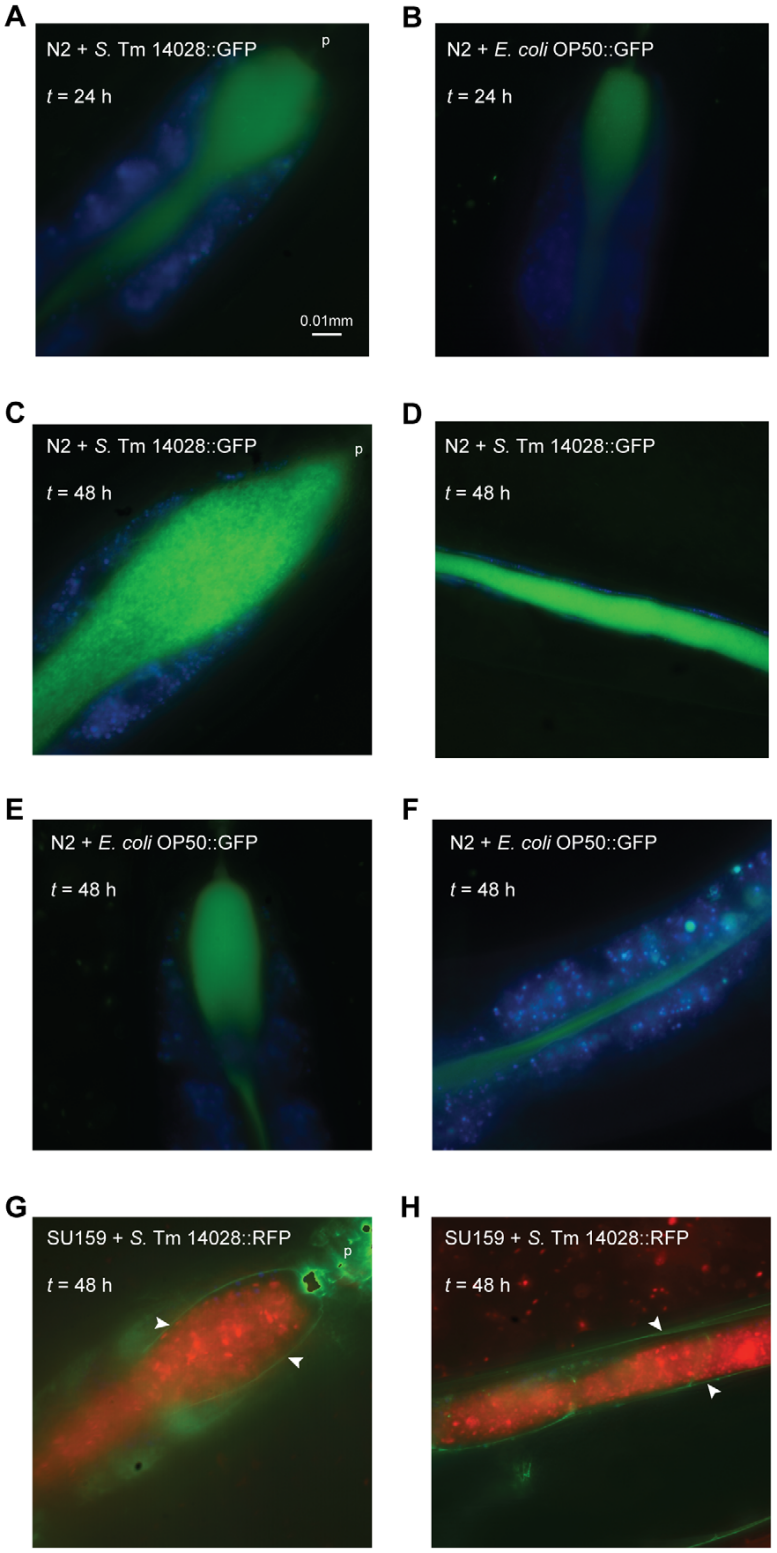
*S*. Typhimurium colonizes the nematode gut and does not disseminate across the intestinal epithelium. (A–F) Wild-type N2 nematodes were fed *S*. Typhimurium (*S*. Tm) 14028::GFP or *E. coli* OP50::GFP. At each time point, live nematodes were harvested and mounted for microscopy in PBS with NaN_3_. In these merged images, *S*. Typhimurium 14028 or *E. coli* OP50 is shown in green and intestinal autofluorescence in blue. (G–H) SU159 nematodes expressing AJM-1::GFP were infected with *S*. Typhimurium 14028::RFP. At each time point, live nematodes were mounted for microscopy. In these merged images, *S*. Typhimurium 14028 is shown in red and both the apical epithelial marker AJM-1 and intestinal autofluorescence in green. The intestinal border is marked by white arrowheads. Images are shown at 100× magnification and the pharynx (p) is indicated. Scale bar represents 0.01 mm. Images are representative of at least 20 nematodes from 3 independent assays.

When observed using fluorescence microscopy 24 h post infection, wild-type nematodes exposed to either GFP-expressing *S*. Typhimurium 14028 or *E. coli* OP50 showed a diffuse fluorescent pattern within the intestinal lumen (Figures 2A and 2B). At later time points though, the fluorescent luminal content expanded substantially in *S*. Typhimurium-infected nematodes and consisted of intact bacterial cells (Figures 2C and 2D). In contrast, nematodes fed on *E. coli* retained a diffuse string-like staining pattern, without significant luminal expansion (Figures 2E and 2F). At no time point of the infection could we detect any significant amount of *S*. Typhimurium bacteria in the surrounding tissues. Here we also provide evidence for the first time, that SU159 nematodes, expressing the GFP-tagged apical epithelial marker AJM-1, displayed a continuous and unbreached staining of the intestinal epithelium upon infection with *S*. Typhimurium (Figures 2G and 2H).

These observations corroborate and extend the observations from previous studies that reported lack of invasiveness of *S*. Typhimurium in *C. elegans* and that in autophagy-competent nematodes, any invading *S*. Typhimurium was observed to be effectively targeted to lysosomes for degradation and thus not significantly detected nor able to cause disruption to the intestinal epithelial cells [39].

### Infection with *S. enterica* evokes a pathogen-specific dissemination of oxidative stress

As ROS plays a protective role during infection of *C. elegans* by *E. faecalis* [35], [36], we hypothesized that the differential virulence of *S*. Typhimurium and *E. coli* in the nematode could relate to their abilities to trigger an oxidative host defense. To test this possibility, we stained wild-type nematodes fed on either non-fluorescent *S*. Typhimurium or *E. coli* with 2′,7′-dichlorodihydrofluorescein diacetate (H_2_DCFDA), a dye that becomes cleaved intracellularly and fluoresces green upon exposure to various intracellular ROS [40], [41].

**Figure 3:**
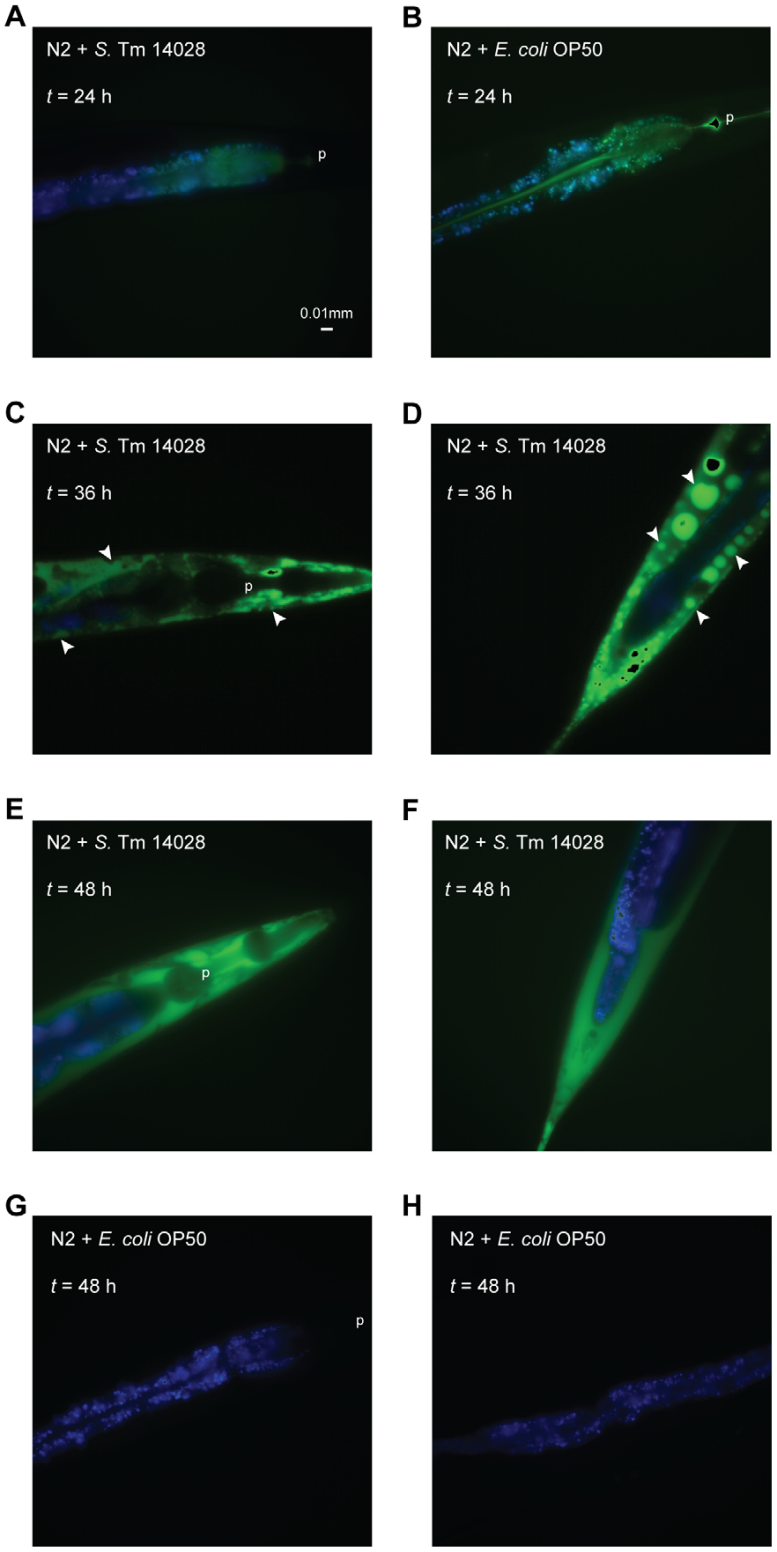
*S*. Typhimurium induces an emergence of ROS from the nematode intestine to the surrounding tissues. (A–H) Wild-type N2 nematodes were fed non-fluorescent *S*. Typhimurium 14028 or *E. coli* OP50. At each time point, nematodes were harvested, stained with H_2_DCFDA to detect intracellular ROS and mounted for microscopy in PBS with NaN_3_. In these merged images, ROS is shown in green and intestinal autofluorescence in blue. The pharynx (p) and ROS foci (white arrowheads) are indicated. Images are shown at 40× magnification and scale bar represents 0.01 mm. Images are representative of at least 20 nematodes from 3 independent assays.

Nematodes fed on either *S.* Typhimurium or *E. coli* revealed green ROS fluorescence clearly contained within the intestinal lumen at 24 h post infection (Figures 3A and 3B). At later time points, *S.* Typhimurium-infected nematodes first exhibited strong green ROS signals contained within foci along the extra-intestinal tissues of the nematode, mainly the hypodermis (Figures 3C and 3D). Subsequently, the signals culminated together in the whole of these tissues (Figures 3E and 3F). In contrast, no escalation or spread of the ROS signal was observed in *E. coli*-fed nematodes at later time points (Figures 3G and 3H).

**Figure 4:**
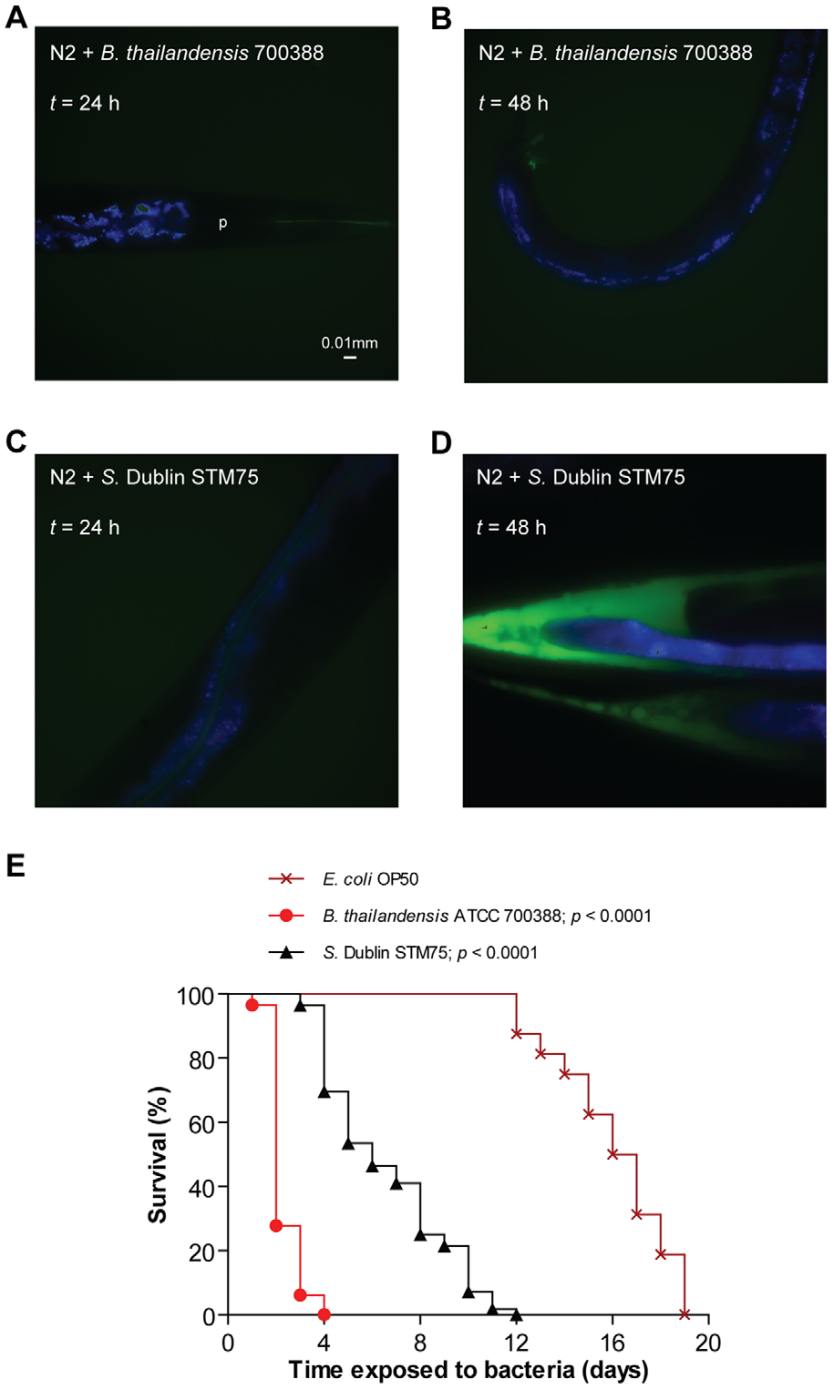
Induction of ROS response is specific to *S. enterica*. (A–D) Wild-type nematodes were infected with non-fluorescent *B. thailandensis* 700388 or *S*. Dublin STM75. At each time point, infected nematodes were harvested, stained with H_2_DCFDA and mounted for microscopy. In these merged images, ROS is shown in green and intestinal autofluorescence in blue. Images are shown at 40× magnification. (E) Survival of wild-type nematodes was compared when fed *E. coli* OP50, *B. thailandensis* 700388 (*p*<0.0001) or *S*. Dublin STM75 (*p*<0.0001; all *p* values as compared to *E. coli*).

To assess whether the dissemination of ROS staining in *S.* Typhimurium-infected nematodes was a generic response to bacterial pathogens or unique to *S.* Typhimurium, we repeated the experiments with two other pathogens, *Burkholderia thailandensis* strain 700388 and *S. enterica* serovar Dublin (*S.* Dublin) strain STM75. In these experiments, the ROS staining pattern appeared rather similar between nematodes fed *B. thailandensis* or *E. coli* (Figures 4A and 4B), despite the fact that *B. thailandensis* infection substantially decreased the lifespan of *C. elegans* (*p*<0.0001, Figure 4E). This was in contrast to infection with *S*. Dublin, which was also pathogenic (*p*<0.0001, Figure 4E), and generated a ROS staining pattern comparable to that caused by *S*. Typhimurium (Figures 4C and 4D). Thus, the emergence of ROS in the extra-intestinal tissues of the nematode appears to be a consequence specific to *S. enterica*.

### Ablation of ROS increases host survival upon infection with *S*. Typhimurium

The above observations indicate that either *C. elegans* relies on a ROS response to defend against selected pathogens such as *S. enterica*, or that the emergence of ROS constitutes a facet of *S. enterica* pathogenicity in the nematode. To distinguish between these possibilities we repeated the infection experiments in the presence of the reductant ascorbic acid [42] or catalase, an enzyme that converts hydrogen peroxide (H_2_O_2_) to water and molecular oxygen [43].

**Figure 5:**
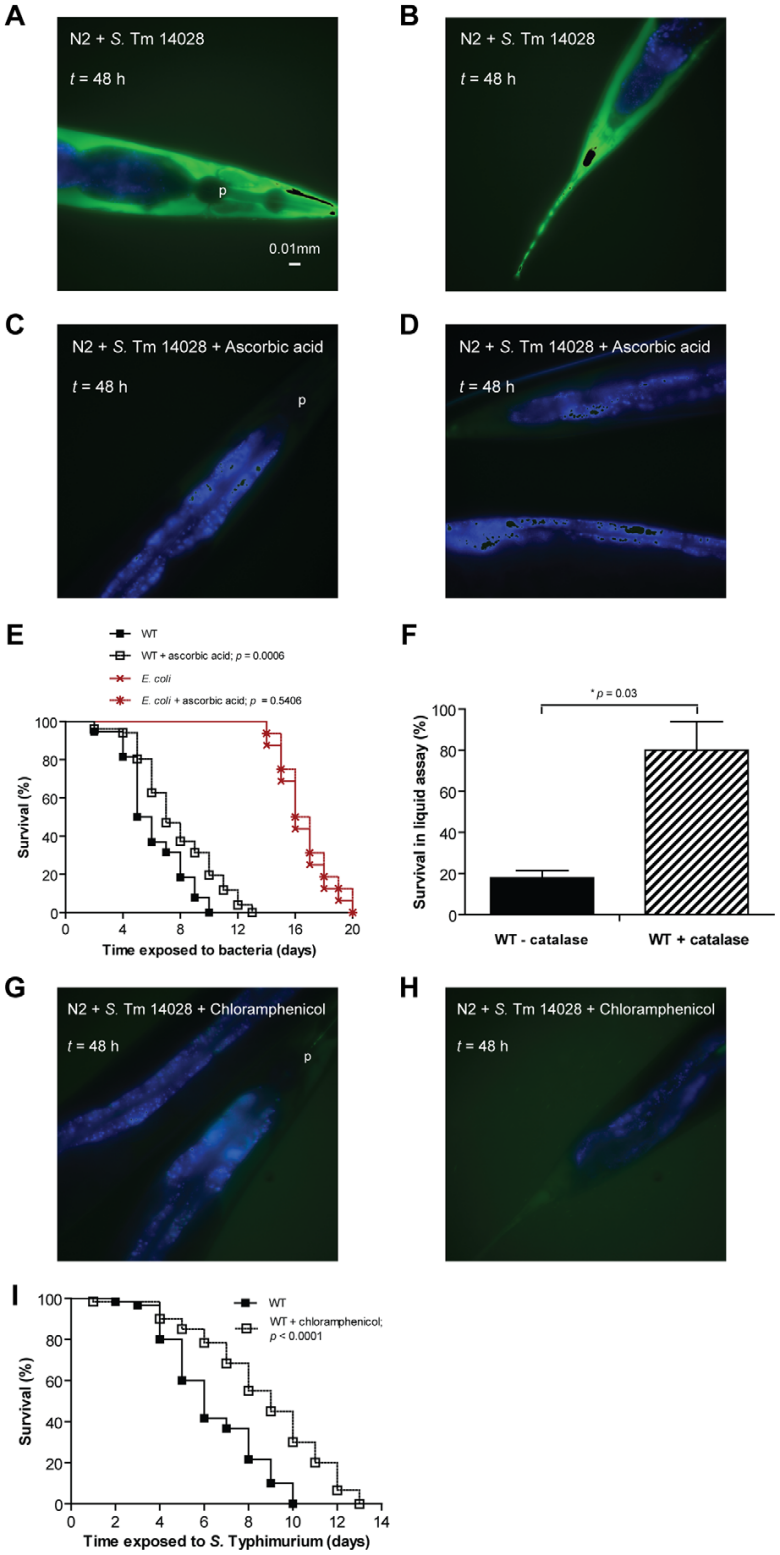
Virulence of *S*. Typhimurium correlates with ROS dissemination in the infected nematode. (A–D) Wild-type nematodes were infected with non-fluorescent *S*. Typhimurium 14028 on NGM agar with or without 50 mM ascorbic acid. At each time point, infected nematodes were harvested, stained with H_2_DCFDA and mounted for microscopy. In these merged images, ROS is shown in green and intestinal autofluorescence in blue. Images are shown at 40× magnification. (E) Survival of wild-type nematodes was compared when fed *E. coli* OP50 (*p* = 0.5406) or *S*. Typhimurium 14028 (WT) (*p* = 0.0006; all *p* values as compared to its respective untreated control) on NGM agar with or without 50 mM ascorbic acid. (F) Survival of wild-type nematodes was compared when infected with *S*. Typhimurium 14028 (WT) in a liquid assay, with or without catalase added (*p* = 0.03). (G–H) Wild-type nematodes were infected with non-fluorescent *S*. Typhimurium 14028 on NGM agar with 10 μg/ml chloramphenicol. At each time point, infected nematodes were harvested, stained with H_2_DCFDA and mounted for microscopy. (I) Survival of wild-type nematodes was compared when infected with *S*. Typhimurium 14028 (WT) on NGM agar with or without 10 μg/ml chloramphenicol (*p*<0.0001).

At a 50 mM concentration of ascorbic acid, nematodes infected with *S.* Typhimurium showed a drastic reduction in the pathogen-induced ROS signal (Figures 5A to 5D) and a significant increase in the lifespan (*p* = 0.0006, Figure 5E). The lifespan of infected nematodes was also enhanced when ascorbic acid was replaced by catalase (*p* = 0.03, Figure 5F). In comparison, neither ascorbic acid nor catalase increased the lifespan of nematodes fed on *E. coli* (*p* = 0.5406, Figure 5E and data not shown).

To investigate if active bacterial protein synthesis was required to trigger the oxidative response, we performed the infection experiments in the presence of the bacterial protein synthesis inhibitor chloramphenicol. Nematodes infected with *S.* Typhimurium in the presence of chloramphenicol also did not reveal any extra-intestinal staining with H_2_DCFDA (Figures 5G and 5H) and consequently, exhibited significantly increased lifespan (*p*<0.0001, Figure 5I).

Combined, these data suggest that the ROS response in *S.* Typhimurium-infected nematodes is detrimental rather than protective against the pathogen.

### Infection-associated ROS dissemination relies on the bacterial thioredoxin 1

**Figure 6:**
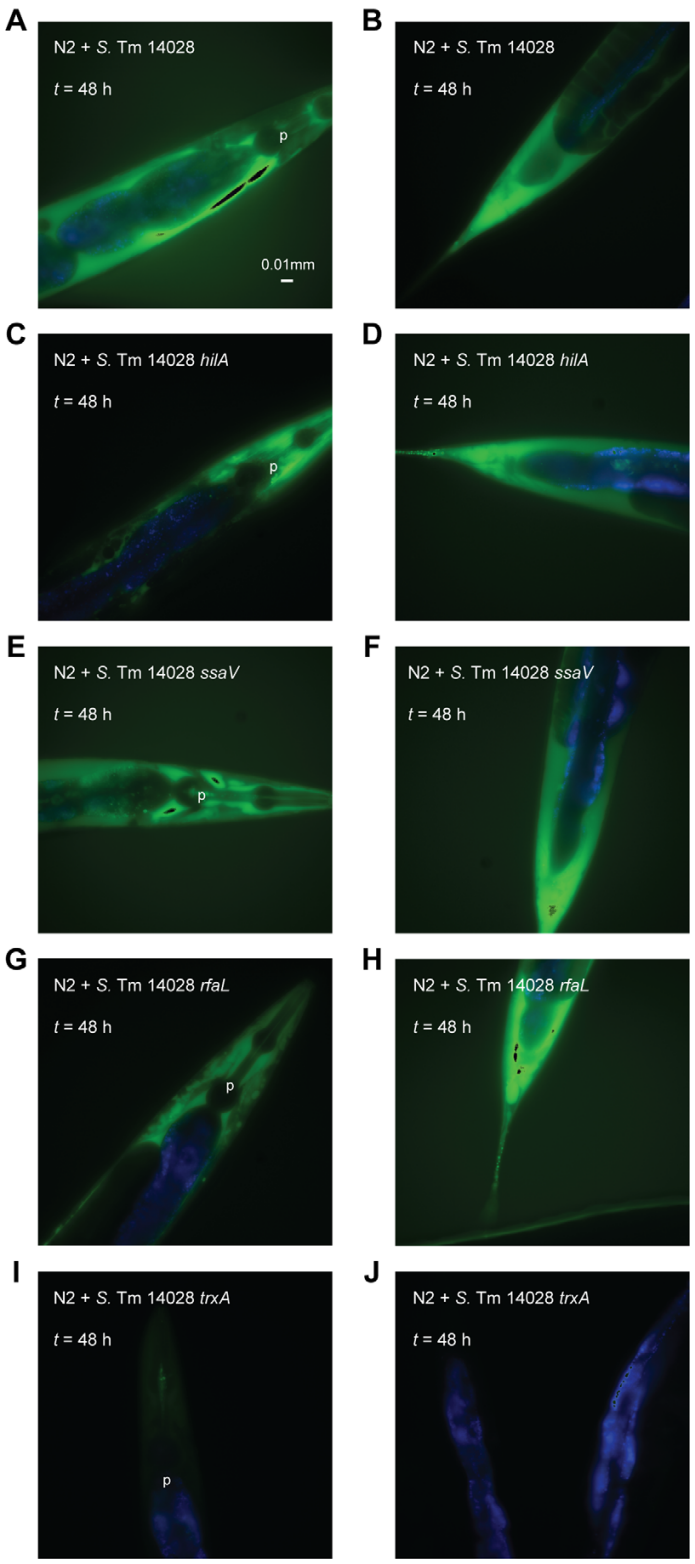
ROS induction in the infected nematode is dependent on the bacterial TrxA. (A–J) Wild-type nematodes were infected with non-fluorescent *S*. Typhimurium 14028 wild-type, *hilA, ssaV, rfaL* or *trxA*. At each time point, infected nematodes were harvested, stained with H_2_DCFDA and mounted for microscopy. In these merged images, ROS is shown in green and intestinal autofluorescence in blue. Images are shown at 40× magnification.

*S. enterica* notably differs from non-pathogenic *E. coli* through its possession of pathogenicity islands such as SPI1 and SPI2. Furthermore, virulence in the murine infection model strongly relies on the expression of many evolutionarily conserved “house-keeping” genes, including those coding for the lipopolysaccharide (LPS) O-antigen and thioredoxin 1 (TrxA) [18], [44]. We thus tested *S*. Typhimurium mutants unable to express SPI1 (*hilA*), SPI2 (*ssaV*), LPS O-antigen (*rfaL*) or thioredoxin 1 (*trxA*) for their ability to induce ROS dissemination in the infected nematode. While the *hilA*, *ssaV* and *rfaL* mutants induced ROS signals comparable to that of the wild-type strain (Figures 6A to 6H), induction of ROS was substantially abrogated in nematodes infected with the *trxA* mutant (Figures 6I to 6J).

### Oxidative stress induction and pathogenesis require the catalytic activity of TrxA

**Figure 7:**
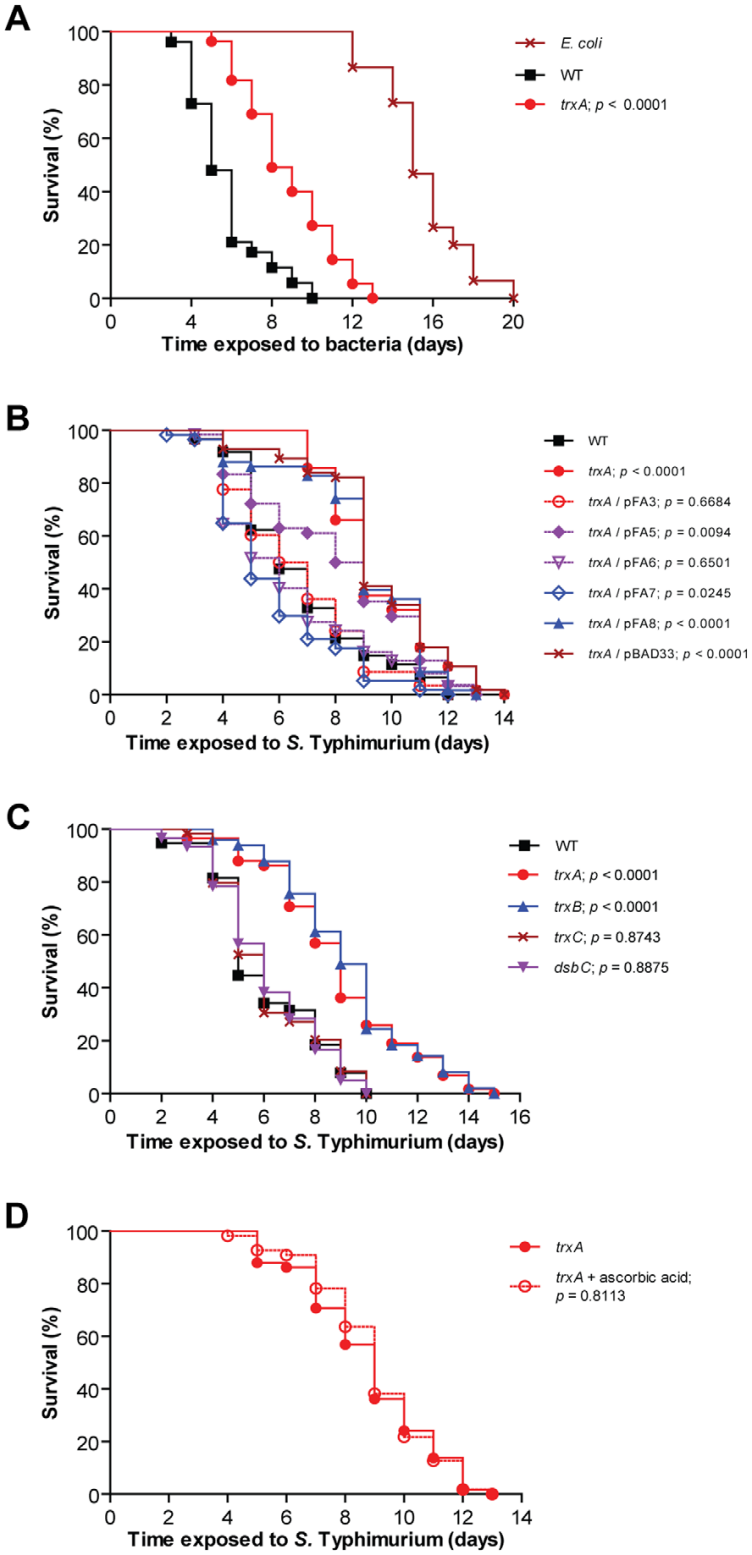
Virulence in the infected nematode relies on the catalytic activity of TrxA. (A) Survival of wild-type nematodes was compared when fed *E. coli* OP50, *S*. Typhimurium 14028 wild-type (WT) or *trxA* (*p*<0.0001 as compared to wild-type). (B) Survival of nematodes was compared when infected with *S*. Typhimurium 14028 wild-type, *trxA* or *trxA trans*-complemented with wild-type TrxA (pFA3), variants of TrxA with decreasing reducing potentials (pFA5-pFA8) or vector (pBAD33) (all *p* values as compared to *S*. Typhimurium 14028 wild-type). (C) Survival of nematodes was compared when infected with *S*. Typhimurium 14028 wild-type, *trxA* (*p*<0.0001), *trxB* (*p*<0.0001), *trxC* (*p* = 0.8743) or *dsbC* (*p* = 0.8875; all *p* values as compared to wild-type). (D) Survival of nematodes was compared when infected with *S*. Typhimurium 14028 *trxA* on NGM agar with or without 50 mM ascorbic acid (*p* = 0.8113).

As the *trxA* mutant did not induce the ROS response in the infected nematode, we compared the lifespans of nematodes infected with wild-type *S*. Typhimurium and the *trxA* mutant. In this experiment, the lifespan of nematodes infected with the *trxA* mutant was significantly prolonged (*p*<0.0001, Figure 7A), further supporting the view that the emergence of ROS in nematodes confers sensitivity to *S*. Typhimurium.

Since the primary structure of TrxA is identical in both *E. coli* and *S.* Typhimurium, observations regarding TrxA in *E. coli* may also be applicable to TrxA in *S.* Typhimurium [18], [45], [46]. In *E. coli* TrxA acts as a cytoplasmic reductant and also indirectly contributes to proper cross-linking of disulphide bonds in the bacterial periplasmic space by providing electrons to disulphide isomerases [46]. In addition, TrxA functions as a protein chaperone independent of its redox activity [47].

To test whether the induction of ROS in *S.* Typhimurium-infected nematodes relied on the redox activity of TrxA, we *trans-*complemented the *trxA* mutant with plasmids coding for wild-type *E. coli* TrxA having a reducing potential (E_o_^r^) of −270 mV (pFA3), or with a series of engineered *trxA* alleles coding for TrxA variants with decreasing reducing potentials (E_o_^r^ with smaller numericals) (pFA5-pFA7) [18], [48]. As a control we used plasmids coding for a catalytically inactive TrxA (pFA8) or the empty cloning vector (pBAD33) (Figure 7B).

We could fully complement virulence by expressing the wild-type TrxA in the *trxA* mutant (*p* = 0.6684). However, full complementation failed when the E_o_^r^ of engineered TrxA variants decreased to −195 mV (pFA5). Accordingly, we did not note any complementation with the catalytically inactive TrxA. Thus, *S*. Typhimurium requires a redox-active TrxA to elicit virulence in *C. elegans*, as reflected by the correlation between the degree of virulence complementation and E_o_^r^.

We also probed the potential contribution of other members of the thioredoxin system, which are homologous to that in *E. coli* [45], [46], by infecting nematodes with *S*. Typhimurium mutants lacking the cytoplasmic thioredoxin reductase TrxB (*trxB*), the cytoplasmic thioredoxin 2 TrxC (*trxC*) or the periplasmic oxidoreductase DsbC (*dsbC*). DsbC reduces and re-oxidizes improper disulphide bonds by receiving electrons from the cytoplasm with the aid of TrxA, TrxB and TrxC [46], [49]. We observed that the *trxB* mutant exhibited attenuated virulence (*p*<0.0001, Figure 7C), similar to the *trxA* mutant. When nematodes were infected with the *trxC* or *dsbC* mutant, we did not see any significant effect on virulence (*p* = 0.8743 and 0.8875 respectively, Figure 7C).

As the *trxA* mutant still appeared more virulent than *E. coli*, yet did not induce the ROS response in the infected host, we tested whether adding ascorbic acid had any effect on the lifespan of nematodes infected with the *trxA* mutant. This regimen, however, did not prolong the lifespan of these nematodes (*p* = 0.8113, Figure 7D).

### Copper chloride restores ROS production and virulence upon infection with the *trxA* mutant

**Figure 8:**
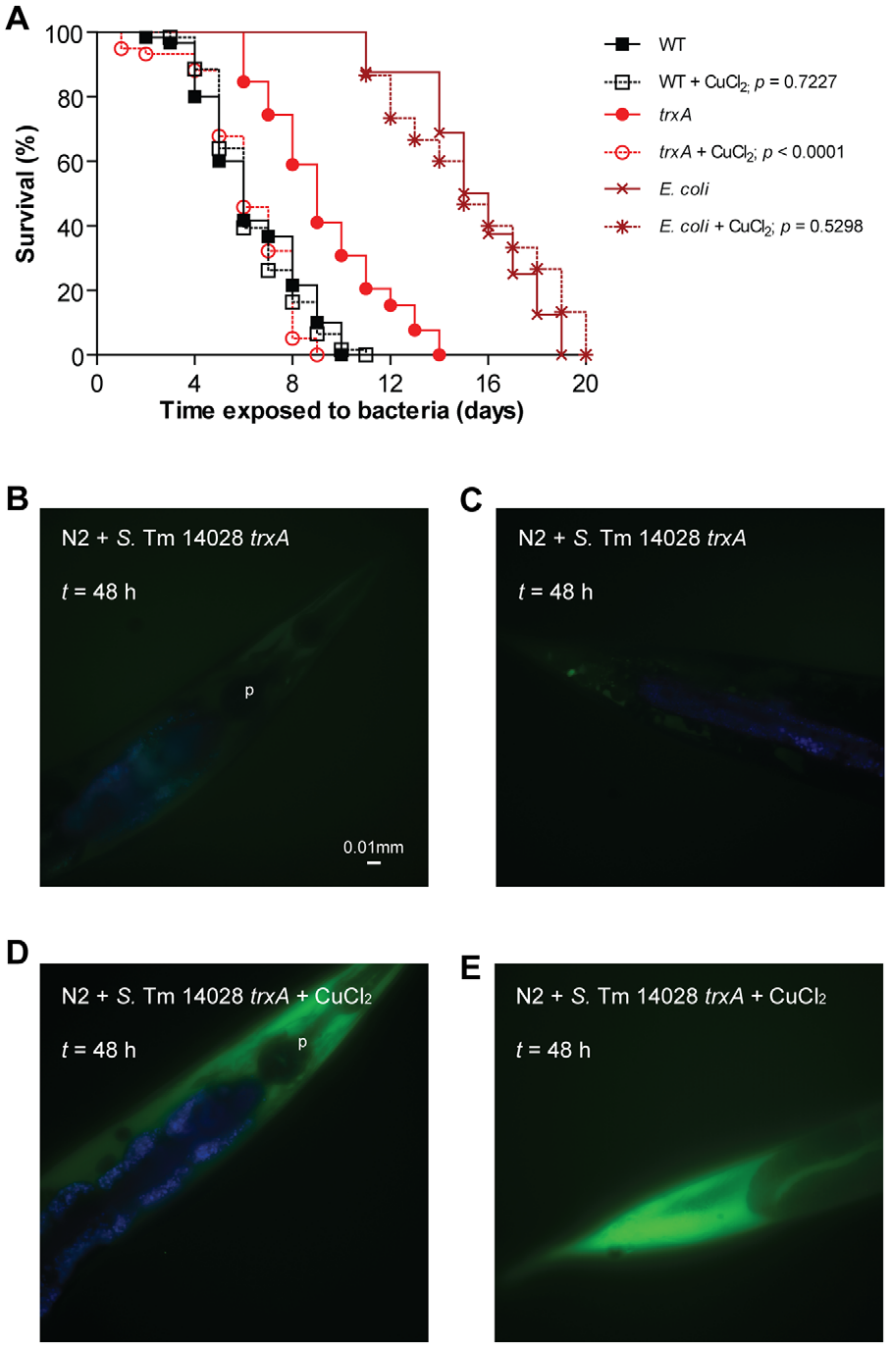
Copper chloride rescues attenuated virulence and lack of ROS induction in *trxA*-infected nematodes. (A) Survival of wild-type nematodes was compared when fed *E. coli* OP50 (*p* = 0.5298), *S*. Typhimurium 14028 wild-type (WT) (*p* = 0.7227) or *trxA* (*p*<0.0001; all *p* values as compared to its respective untreated control), preincubated with or without 2 mM CuCl_2_. (B–E) Wild-type nematodes were infected with non-fluorescent *S*. Typhimurium 14028 *trxA*, preincubated with or without 2 mM CuCl_2_. At each time point, infected nematodes were harvested, stained with H_2_DCFDA and mounted for microscopy. In these merged images, ROS is shown in green and intestinal autofluorescence in blue. Images are shown at 40× magnification.

Copper chloride (CuCl_2_) catalyzes disulphide bond formation for periplasmic *E. coli* proteins *in vivo* [49]. To perturb periplasmic disulphide bond formation, we fed *C. elegans* with *E. coli,* wild-type *S.* Typhimurium or the *trxA* mutant that had been pre-exposed to a sublethal 2 mM concentration of CuCl_2_. Feeding *C. elegans* with *E. coli* (*p* = 0.5298) or wild-type *S*. Typhimurium (*p* = 0.7227) pre-exposed to CuCl_2_ did not affect the lifespan of the nematodes, as compared to unexposed bacteria (Figure 8A). Surprisingly, infection with the *trxA* mutant pre-treated with CuCl_2_ caused a significant shortening of the lifespan of the nematodes (*p*<0.0001, Figure 8A), comparable to that of nematodes infected with wild-type *S.* Typhimurium. In parallel we also noted restoration of the ROS staining in *trxA*-infected nematodes upon CuCl_2_ treatment (Figures 8B to 8E).

## Discussion

In this study, we described the emergence of ROS as a key event in the infection pathogenesis of *S. enterica* in the soil nematode *C. elegans*. As evidenced by staining infected nematodes with the oxidative stress indicator H_2_DCFDA, we observed that ROS were initially confined to the intestinal lumen and subsequently appeared in the surrounding tissues. Still, *S.* Typhimurium remained in the intestinal lumen of the nematode throughout the infection. This phenomenon was not a general response to bacteria, as it did not occur in nematodes fed on *E. coli* or the nematocidal *B. thailandensis*.

Treatment of *S*. Typhimurium-infected nematodes with the bacterial protein synthesis inhibitor chloramphenicol not only prolonged their lifespan, but also resulted in ablation of the extra-intestinal ROS signals. This suggests that replication of *S.* Typhimurium, or a bacterial factor being actively induced during infection, triggered the ROS response. Production of ROS is considered a key facet of host antimicrobial responses [45]. However, while the use of the reductant ascorbic acid or H_2_O_2_-degrading catalase in our subsequent experiments counteracted the emergence of ROS, these regimens extended rather than shortened the lifespan of infected nematodes. This strongly implies that the ROS response did not act to protect *C. elegans* from *S. enterica* infection, unlike that observed with *E. faecalis*-infected nematodes [30], [31]. Rather, the *S. enterica-*induced ROS mediated the infection pathogenesis. This is reminiscent of endotoxin-induced septic shock in rodents, where ROS have been suggested to be the mediators of the symptoms, and where the severity of disease can be eliminated by systemic application of ascorbic acid [34], [50]. Combined, these observations implicate excessive ROS production as an effector of severe infection pathogenesis across a broad evolutionary spectrum of hosts.

When *C. elegans* were infected with *E. faecalis*, ROS were generated to protect the host and the addition of a NADPH oxidase inhibitor dampened this response [36], suggesting that members of the NOX/DUOX protein family may contribute to the phenomenon [51]. Subsequent studies revealed that the protective ROS induced during an *E. faecalis* infection was mediated by the dual oxidase Ce-DUOX/BLI-3 [35]. When we investigated the role of Ce-DUOX/BLI-3 in *S.* Typhimurium infection by reducing *bli-3* expression with RNA interference, we did not observe any significant contribution by the dual oxidase towards the induced ROS response (data not shown). Apart from the contrasting roles ROS play during the two infections, there is also the possibility that there are other NOX/DUOX protein members or even other non-related protein families responsible for generating H2DCFDA-detectable ROS in the nematode during a *S. enterica* infection.

In fact, H_2_DCFDA lacks substrate specificity and fluoresces in the presence of a wide variety of ROS including H_2_O_2_, superoxide anions, nitric oxide, peroxyl and hydroxyl radicals [52]. H_2_DCFDA also has a spontaneous tendency to photo-oxidize if left for long periods of incubation [52], although in our series of experiments this problem was clearly circumvented with *E. coli* and *B. thailandensis*-infected nematodes as controls. Still, that catalase prolonged nematode lifespan upon *S*. Typhimurium infection strongly implicated H_2_O_2_ as one of the mediators of *S*. Typhimurium pathogenesis in *C. elegans*. After all, H_2_O_2_ stands as a cellular endpoint metabolite derived from several reactive intermediates; free iron in the living system unavoidably also participates in redox cycling to generate ROS [41], [52]. Thus free radical reactions resulting in significantly detectable green signals in our experiments are likely more complex than understood. Hence, the *S. enterica*-induced infection response in *C. elegans* encompasses, without doubt, the emergence of ROS which may consist of H_2_O_2_ *per se* or of reactive intermediates which have broken down into H_2_O_2_. Probes specialized for detecting individual species of ROS, such as the Griess reagent for nitrite compounds or dihydroethidum derivatives for superoxide anions, could be included in further studies to better delineate this *S. enterica*-induced phenomenon.

Clearly, while important, the ROS response did not stand as the sole mechanism mediating pathogenesis in *S. enterica-*infected nematodes. This notion emerges from our observations that *i*) a *trxA* mutant was still more virulent than non-pathogenic *E. coli*; and *ii*) a LPS O-antigen deficient *rfaL* mutant, also attenuated in virulence in the nematode (data not shown), still evoked a ROS response similar to wild-type *S.* Typhimurium. Likewise, mutational depletion of key *Salmonella* virulence factors, such as the SPI1 and SPI2 T3SS, did not affect the ROS induction. In contrast, loss of the bacterial virulence-associated “house-keeping” gene *trxA* resulted in an ablation of the ROS response, and substantially restored nematode lifespan.

TrxA is a small evolutionarily conserved reductase. It possesses a high capability of reducing cytoplasmic proteins, and via its association with periplasmic disulphide isomerases, allows for the repair of periplasmic proteins with aberrantly oxidized sulphydryl groups [40]. Interestingly, TrxA in itself also has the potential to behave as an immune signaling molecule when released from the bacterial cytoplasm [53], and hence has the possibility to directly mediate the dissemination of ROS. However, since the primary structure of TrxA is identical in both *S.* Typhimurium and *E. coli* [45], [46], possession of TrxA alone clearly is not sufficient to confer *E. coli* the ROS-inducing ability when infecting nematodes. In accordance, *S.* Typhimurium TrxA is co-induced with SPI2 and needed for the proper activity of SPI2 during infection of murine models [17]. However, the fact that a SPI2 mutant still evoked a ROS response in *C. elegans* suggests that the virulence input by TrxA in the nematode infection model relies uniquely on some other *Salmonella*-specific factors other than SPI2.

Two lines of data implicate redox catalysis as an important characteristic of TrxA in mediating pathogenesis in *C. elegans*. First, the *trxA* mutant was fully *trans*-complemented with wild-type TrxA and genetically engineered TrxA variants, however only with those having an E_o_^r^ greater than −195 mV. Second, we could substitute TrxA simply by pre-exposing the *trxA* mutant to sublethal concentrations of CuCl_2_. As CuCl_2_ is known to catalyze disulphide bond formation for periplasmic proteins in *E. coli* [49], our observations would connect the ROS-associated pathogenesis to periplasmic disulphide bond formation. Still, we could not attenuate virulence or ROS formation by mutating TrxC or DsbC. This suggests that attenuation of the *trxA* mutant in *C. elegans* does not relate to a general defect in periplasmic disulphide bond formation.

The Gram-positive bacterium *Streptococcus pneumoniae*, a natural human pathogen, produces an exceptionally high concentration of H_2_O_2_ *in vitro* [54]. Furthermore, this ability has been associated with its virulence in *C. elegans* [55], [56]. Still, we failed to demonstrate significant H_2_O_2_ production by wild-type *S*. Typhimurium or the *trxA* mutant *in vitro.* In addition, we also did not observe any hypodermal ROS staining in nematodes infected with the virulent *Str. pneumoniae* serotype I strain BHN32 (data not shown), providing evidence that H_2_O_2_ produced by the pathogen is unlikely involved in this *S. enterica-*induced ROS response. This idea was also further supported by the initial appearance of ROS signals as independent foci in the hypodermis, despite the lack of any detectable *S. enterica* at these sites. Our data thus favor the hypothesis in which *C. elegans* mounts an overwhelming pathogen-specific ROS response in the hypodermis upon infection with *S. enterica,* reminiscent to infection-associated multi-organ failure or septic shock caused by innate immune responses in higher vertebrates [32]–[34].

## Materials and Methods

### Nematode and Bacteria Strains

Nematode and bacteria strains used in this study are listed in Table S1. Nematode strains were cultured and maintained at 20°C on modified nematode growth media (NGM, 0.35% peptone) agar and fed with *E. coli* strain OP50, as described [19]. Bacteria strains were grown in Luria-Bertani (LB) broth at 37°C. When necessary, LB broth is supplemented with ampicillin 100 μg/ml; chloramphenicol 10 μg/ml; kanamycin 50 μg/ml and/or L-arabinose 5% (w/v) (Sigma-Aldrich, St. Louis, MO).

### Survival Assays

Bacterial strains were grown overnight in LB broth at 37°C and lawns were prepared by spreading 200 μl of overnight culture on modified NGM agar. 20 L4-staged wild-type N2 nematodes were subsequently infected as described [57]. Briefly, nematodes were set down on unseeded agar before transferring to bacterial lawns to reduce, as much as possible, the transfer of *E. coli.* No visible *E. coli* growth on pathogenic lawns was observed nor was there any crowding of nematodes. Nematode survival was scored at 24°C and nematodes were considered dead upon failure to respond to gentle touch by a platinum wire. Nematodes were also transferred to fresh bacterial lawns every day. Results are representative of 3 independent assays, each with triplicates.

For experiments involving L-arabinose, ascorbic acid and chloramphenicol, modified NGM agar was impregnated with each of these compounds at the following concentration to ensure maximum exposure: L-arabinose 5% (w/v); ascorbic acid 50 mM (Sigma-Aldrich); chloramphenicol 10 μg/ml.

For experiments involving CuCl_2_, *E. coli* and S. Typhimurium strains were grown overnight in LB broth supplemented with or without 2 mM CuCl_2_ before seeding on modified NGM agar.

### Immunofluorescence Assays

Fluorescent *E. coli* and *S.* Typhimurium lawns were prepared by spreading 200 μl of overnight LB culture on modified NGM agar. L4-staged N2 or SU159 nematodes were added to these lawns and infected as described above. At each time point, live nematodes were harvested and mounted for microscopy in phosphate-buffered saline (PBS) with 25 mM sodium azide (NaN_3_; Sigma-Aldrich). Slides were visualized on a LEICA DMRE microscope and images were analyzed by GNU Image Manipulation Program (version 2.6.3). Images are representative of at least 20 nematodes from 3 independent assays.

### Reactive Oxygen Species Detection

2′,7′-dichlorodihydrofluorescein diacetate (H_2_DCFDA; Sigma-Aldrich) is used to visualize intracellular ROS in nematodes. 2 mM stock aliquots of H_2_DCFDA were prepared in dimethyl sulfoxide and stored in the dark at −80°C. L4-staged N2 nematodes were infected as described above. At each time point, infected nematodes were harvested into tubes and washed twice with M9 buffer. Nematodes were subsequently incubated with 25 μM H_2_DCFDA in 250 μl M9 buffer, in the dark for 30 min in a 20°C water bath. After staining, nematodes were washed thrice with M9 buffer and mounted for microscopy in PBS with 25 mM NaN_3_. Slides were visualized on a LEICA DMRE microscope and images were analyzed by GNU Image Manipulation Program. Images are representative of at least 20 nematodes from 3 independent assays.

### Liquid Infection Assays with Catalase

*E. coli* and *S.* Typhimurium strains were grown overnight in LB broth at 37°C. The overnight culture was resuspended in S Basal liquid media and 800 μl of the suspension was aliquoted into each well of a 48-well flat-bottomed plate. 190 U of catalase (Sigma-Aldrich) was added into each well and mixed thoroughly. 20 L4-staged N2 nematodes were transferred to each well, incubated at 24°C for 24 h and examined with a light microscope. Nematodes were considered dead when neither body twitching nor pharyngeal pumping could be observed. Infected nematodes were also transferred to unseeded agar to verify the phenotype. Results are representative of 3 independent assays, each with triplicates.

### Statistical Analysis

Survival curves were analyzed using the PRISM (version 5.0) software. Kaplan–Meier estimation of survival curves with *p* values <0.05 were considered significantly different from the control. Student's *t* test was used to analyze the results from the liquid infection assays.

## Supporting Information

Table S1 **List of nematode and bacteria strains used in this study.**
(PDF)
