# Supplementary material for: Pathogenicity of Salmonella enterica in Caenorhabditis elegans Relies on Disseminated Oxidative Stress in the Infected Host
Source: PLoS One. 2012 Sep 28;7(9):e45417. doi: 10.1371/journal.pone.0045417 (PMC3461013; doi:10.1371/journal.pone.0045417)
Supplement: Table S1 — List of nematode and bacteria strains used in this study. (PDF) [file pone.0045417.s001.pdf]

Table S1. List of nematode and bacteria strains used in this study

Strain		Genotype or description	Source or reference
**Nematode**			
C. elegans	N2	Wild-type variant Bristol	CGC[a]
	SU159	*ajm-1(ok160) X; jcEx44 [pJS191 (ajm-1::gfp) + pRF4 (rol-6(su1006)]*	CGC[a]
**Bacteria**			
S. Typhimurium	ATCC 14028	Wild-type 14028 strain	ATCC[b]
	ATCC 14028::GFP	Wild-type 14028 *proU::gfp*, chloramphenicol^r	MR Lab, [1]
	ATCC 14028::RFP	Wild-type 14028 + pMW635, araC, ampicillin^r	MR Lab, Oliver Pabst
	hilA	ATCC 14028 *hilA*, kanamycin^r	[2]
	ssaV	ATCC 14028 non-polar *ssaV*, chloramphenicol^r	[3]
	rfaL	ATCC 14028 *rfaL*, chloramphenicol^r	[4]
	trxA	ATCC 14028 non-polar *trxA*	[4]
	trxA/pFA3	*trxA* + wild-type TrxA, $E_o{}^r$ -270 mV, araC, chloramphenicol^r	[4,5]
	trxA/pFA5	*trxA* + Grx-type TrxA, $E_o{}^r$ -195 mV, araC, chloramphenicol^r	[4,5]
	trxA/pFA6	*trxA* + DsbA-type TrxA, $E_o{}^r$ -204mV, araC, chloramphenicol^r	[4,5]
	trxA/pFA7	*trxA* + PDI-type TrxA, $E_o{}^r$ -221 mV, araC, chloramphenicol^r	[4,5]
	trxA/pFA8	*trxA* + non-catalytic TrxA, araC, chloramphenicol^r	[4,5]
	trxA/pBAD33	*trxA* + empty vector, araC, chloramphenicol^r	[4,5]
	trxB	ATCC 14028 *trxB,* kanamycin^r	[4]
	trxC	ATCC 14028 *trxC,* kanamycin^r	[4]
	dsbC	ATCC 14028 *dsbC,* kanamycin^r	[4]
S. Dublin	STM75	Wild-type STM75 strain	[6]
	STM75::RFP	Wild-type STM75 + pMW635, araC, ampicillin^r	MR Lab, Oliver Pabst
E. coli	OP50	Laboratory food source	CGC[a]
	OP50::GFP	OP50 + pFVP25.1, ampicillin^r	CGC[a]
B. thailandensis	ATCC 700388	Wild-type reference strain isolated from Thailand	ATCC[b]

[a] Caenorhabditis Genetics Center, University of Minnesota, Minneapolis, U.S.A.

[b] American Type Cell Collection, Virginia, U.S.A.

REFERENCES

1. Hautefort I, Proença MJ, Hinton JCD (2003) Single-copy green fluorescent protein gene fusions allow accurate measurement of *Salmonella* gene expression in vitro and during infection of mammalian cells. Appl Environ Microbiol 69: 7480-7491.
2. Negrea A, Bjur E, Ygberg SE, Elofsson M, Wolf-Watz H, et al. (2007) Salicylidene acylhydrazides that affect type III protein secretion in *Salmonella enterica* serovar Typhimurium. Antimicrob Agents Chemother 51: 2867-2876.
3. Shea JE, Beuzon CR, Gleeson C, Mundy R, Holden DW (1999) Influence of the *Salmonella* Typhimurium pathogenicity island 2 type III secretion system on bacterial growth in the mouse. Infect Immun 67: 213-219.
4. Bjur E, Eriksson-Ygberg S, Aslund F, Rhen M (2006) Thioredoxin 1 promotes intracellular replication and virulence of *Salmonella enterica* serovar Typhimurium. Infect Immun 74: 5140-5151.
5. Mössner E, Huber-Wunderlich M, Rietsch A, Beckwith J, Glockshuber R, et al. (1999) Importance of redox potential for the *in vivo* function of the cytoplasmic disulfide reductant thioredoxin from *Escherichia coli*. J Biol Chem 274.
6. Tezcan-Merdol D, Eriksson Ygberg S, Rhen M (2007) The *Salmonella enterica* virulence plasmid and the spv gene cluster. In: Rhen M, Maskell D, Mastroeni P, Therfall J, editors. *Salmonella*: molecular biology and pathogenesis. Norfolk: Horizon Bioscience. pp. 89-103.
